# Optical modelling data for room temperature optical properties of organic–inorganic lead halide perovskites

**DOI:** 10.1016/j.dib.2015.03.004

**Published:** 2015-03-28

**Authors:** Yajie Jiang, Martin A. Green, Rui Sheng, Anita Ho-Baillie

**Affiliations:** Australian Centre for Advanced Photovoltaics (ACAP), School of Photovoltaic and Renewable Energy Engineering, University of New South Wales, Sydney 2052, Australia

## Abstract

The optical properties of perovskites at ambient temperatures are important both to the design of optimised solar cells as well as in other areas such as the refinement of electronic band structure calculations. Limited previous information on the optical modelling has been published. The experimental fitting parameters for optical constants of CH_3_NH_3_PbI_3−*x*_Cl_*x*_ and CH_3_NH_3_PbI_3_ perovskite films are reported at 297 K as determined by detailed analysis of reflectance and transmittance data. The data in this study is related to the research article “Room temperature optical properties of organic–inorganic lead halide perovskites” in Solar Energy Materials & Solar Cells [1].

**Specifications table**

**Table:** 

Subject area	*Physics*
More specific subject area	*Photovoltaics*
Type of data	*Table, figure*
How data was acquired	*J.A. Woollam M-2000 Spectroscopic Ellipsometer; Varian Cary UV–vis–NIR spectrophotometer*
Data format	*Analysed*
Experimental factors	*Ellipsometry data and T were obtained for borosilicate glass, TiO*_*2*_*on glass and TiO*_*2*_*coated glass in the 0.8–3* *eV.*
Experimental features	*WVASE*^®^*is used to model the optical properties of the individual borosilicate glass and TiO*_*2*_*layer based on the ellipsometry and transmission data for these layers. The parameters are then fed into the model for the multi-layer stack to develop the optical model for the perovskite.*
Data source location	*School of Photovoltaic and Renewable Energy Engineering University of New South Wales, Sydney, Australia*
Data accessibility	*Data is with this article*

**Value of the data**

• The fabrication processes of each sample were explained.
• The surface morphology of each individual layer was characterised.
• The data provides optical analysis of all layers in the perovskite sample under study ensuring the accuracy of the modelling.
• Parameters for optical model of each material were presented.

## 1 Data, experimental design, materials and methods

The fabrication details of CH_3_NH_3_PbI_3_ thin film at room temperature are presented with surface morphology characterised by Atomic Force Microscopy. The optical properties of each individual layer were investigated by variable angle spectroscopic ellipsometry and spectrophotometry. In order to determine the optical properties of CH_3_NH_3_PbI_3_ thin film, the optical properties from any substrate components were studied first. Different optical models were built to deduce the optical parameters of each layer.

## 2 Material preparation

The CH_3_NH_3_PbI_3_ film was deposited onto a borosilicate glass substrate coated with 45 nm thick compact TiO_2_ layer using a relatively standard sequential solution processing technique as previously reported [2].

## 3 Cleaning of borosilicate glass substrate

Borosilicate glass substrates (2.5×2.5 mm^2^) were cleaned in 2% Hallmanex detergent, acetone and Isopropanol in ultrasonic bath for 10 min in each cleaning agent followed by oxygen plasma treatment for 10 min.

## 4 TiO_2_ compact layer deposition

Compact TiO_2_ layer was deposited by spin-coating a mildly acidic solution of titanium isopropoxide in ethanol at 2500 rpm for 60 s on glass followed by anneal at 500 °C for 30 min.

## 5 Perovskite film fabrication

The CH_3_NH_3_PbI_3_ film was fabricated using sequential solution processing technique as previously reported [2] on borosilicate glass substrate coated with 45 nm compact TiO_2_ layer. Methylammonium iodide (MAI) was synthesised following a previously reported method [3] by reacting 24 mL of 0.20 mol methylamine (33 wt% in absolute ethanol, Aldrich), 10 mL of 0.04 mol hydroiodic acid (57 wt% in water, Aldrich) and 100 mL ethanol in a 250 mL round bottom flask at 0 °C for 2 h with stirring. After the reaction, the precipitate was recovered by a vacuum evaporator at 60 °C and then dissolved in ethanol followed by sedimentation in diethyl ether until the white MAI powder appears. The final product was collected and dried at 60 °C in an oven and dehydrated in a vacuum chamber. PbI_2_ was purchased from Sigma-Aldrich and dissolved as received in N, N-dimethylformamidthe (DMF) with a concentration of 462 mg/ml under stirring at 70 °C. Perovskite was deposited by spin-coating PbI_2_ solution at 2000 rpm for 60 s, followed by annealing at 70 °C for 30 min after cooling down, the film was dipped into MAI solution dissolved in a 2-propanol (10 mg/ml) for 20 s and then annealed at 100 °C for 10 min.

## 6 Surface roughness characterisation

**Fig. 1:**
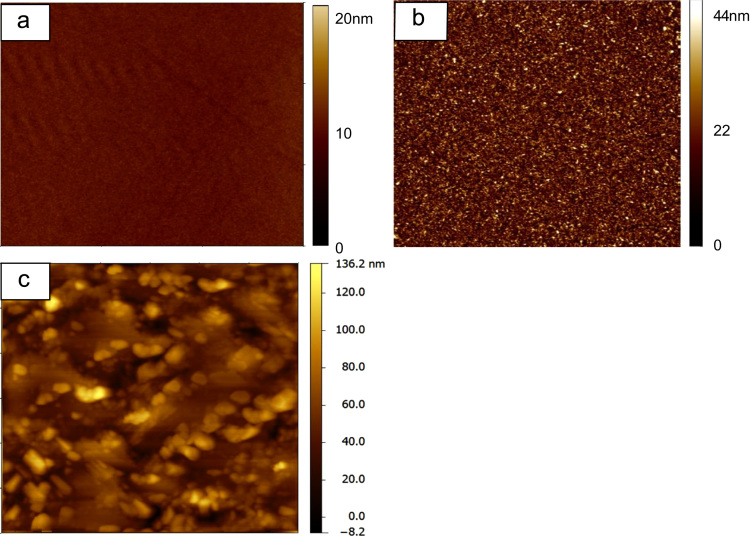
Atomic Force Microscopy top view images of (a) glass substrate; (b) TiO_2_; (c) CH_3_NH_3_PbI_3_ thin film.

The surface morphology was characterised by Atomic Force Microscopy (AFM). The top view of glass, TiO_2_ compact layer and CH_3_NH_3_PbI_3_ thin film AFM images are shown in Fig. 1. The surface roughness of glass and TiO_2_ compact layer is negligible compared with CH_3_NH_3_PbI_3_ thin film. The median surface roughness of CH_3_NH_3_PbI_3_ thin film is 50 nm and has been considered in the optical modelling, included as error bars representing measurement uncertainty.

## 7 Optical measurement and modelling

Ellipsometry was carried out using a J.A. Woollam M-2000 Spectroscopic Ellipsometer in the wavelength range of 370–1690 nm. All ellipsometry data in this study are collected from three incident angles 45°, 50° and 55°. The reflection (*R*) and transmission (*T*) measurements were carried out using a Varian Cary UV–vis–NIR spectrophotometer at normal incidence. Ellipsometry data and *T* were obtained for borosilicate glass and TiO_2_ on glass in the 0.8–3 eV. *R* for the borosilicate glass and TiO_2_/Glass are not required, as the ellipsometry has been carried out in reflection mode and *T* in the 1–4 eV range provides complementary information due to the different path length the light travels in the sample.

WVASE^®^ is used to model the optical properties of the individual borosilicate glass and TiO_2_ layer based on the ellipsometry and transmission data for these layers [4]. The parameters are then fed into the model for the multi-layer stack to develop the optical model for the perovskite.

## 8 Glass substrates

**Fig. 2:**
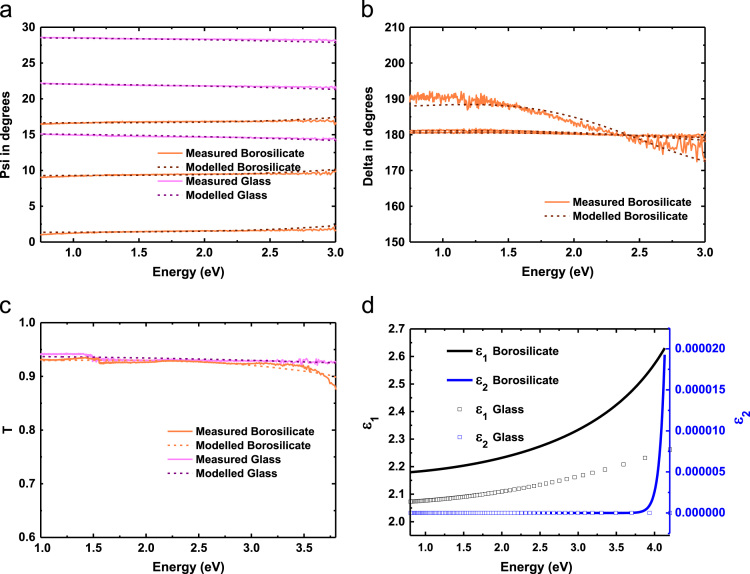
Modelled (dash lines) and experimental (solid lines) (a) amplitude component *Ψ*; (b) phase difference *Δ*; (c) transmission *T*; (d) real (*ε*_1_) and imaginary (*ε*_2_) parts of dielectric constants of borosilicate glass and glass substrates (except phase difference *Δ* for glass not shown as it is zero for the whole wavelength range).

Cauchy model and two Gaussian dispersions (see Table 1 for parameters) were used to model the absorption of the 2.8 mm thick borosilicate glass (to be later used for the CH_3_NH_3_PbI_3_ film modelling) and the second glass substrate (to be later used for the CH_3_NH_3_PbI_3−*x*_Cl_*x*_ film modelling) in the transparent region and above 3 eV respectively. Fig. 2(a); (b); and (c) shows the experimental and modelled amplitude component *Ψ*; phase difference *Δ*; transmission *T* of the borosilicate glass and the second glass substrate (except phase difference *Δ* for glass not shown as it is zero for the whole wavelength range) respectively which are used to determine the real (*ε*_1_) and imaginary (*ε*_2_) parts of its dielectric constants, see Fig. 2(d). A surface SiO_2_ layer on the bottom of 2.8 mm thick borosilicate glass has been modelled to be 106 nm thick using optical constants from Palik [5].

## 9 TiO_2_ compact layer on glass

**Fig. 3:**
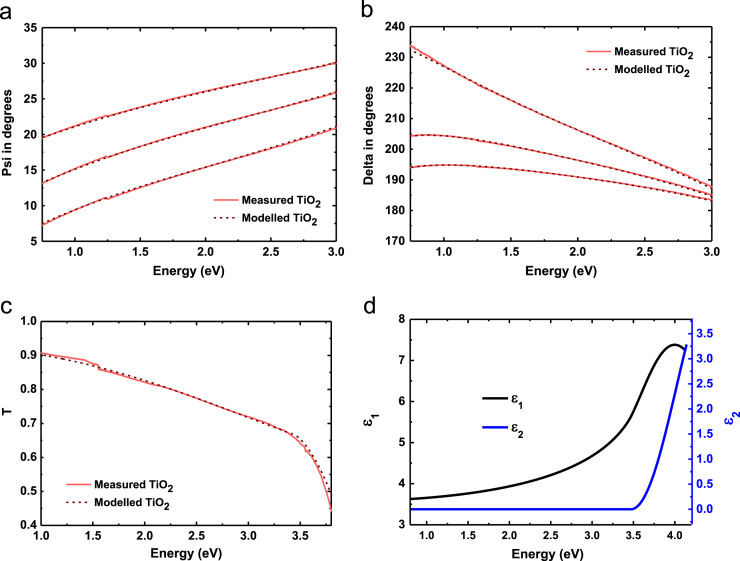
Modelled (red dash lines) and experimental (red solid lines) (a) amplitude component *Ψ*; (b) phase difference *Δ*; (c) transmission *T*; and (d) real (*ε*_1_) and imaginary (*ε*_2_) parts of dielectric constants of TiO_2_ thin film on borosilicate glass substrate.

Tauc–Lorentz dispersion (see Table 2 for parameters) has been used to model the TiO_2_ thin film by combining Tauc bandedge with Lorentz broadening function [6]. The fittings and corresponding optical constants are shown in Fig. 3. Fig. 3(a); (b); and (c) shows the experimental and modelled amplitude component *Ψ*; phase difference *Δ*; transmission *T* of the TiO_2_ on borosilicate glass respectively which are used to determine the real (*ε*_1_) and imaginary (*ε*_2_) parts of its dielectric constants, see Fig. 3(d). The TiO_2_ film thickness has been determined to be 44 nm.

## 10 Perovskite

The optical properties and films thicknesses of borosilicate glass and TiO_2_ layer were extracted and were fixed in the simulation of CH_3_NH_3_PbI_3_ perovskite properties. Two Psemi-Triangle (PSTRI) oscillators were used to describe the electronic transitions at absorption peaks, and Gaussian oscillators were used for the other regions. While for vapour-deposited CH_3_NH_3_PbI_3−*x*_Cl_*x*_ perovskite film on glass by Wehrenfennig, optical properties of glass (measured and modelled in previous section) with a thickness of 1.7 mm [7] were used to model the CH_3_NH_3_PbI_3−*x*_Cl_*x*_ perovskite properties. The *R* and *T* were digitised from literature between 0.8–2.5 eV, and two PSTRI oscillators were able to reproduce the experimental results in this range without the use of other oscillators at higher energy. Tables 3 and 4 list the parameters used for modelling *ε*_2_. *A*, *E* and *B* represent the amplitude, centre energy and broadening of each oscillator respectively. WL and WR stand for the endpoint positions relative to centre energy position, while AL and AR are the relative magnitudes of the left and right control points compared to amplitude (*A*).

**Fig. 4:**
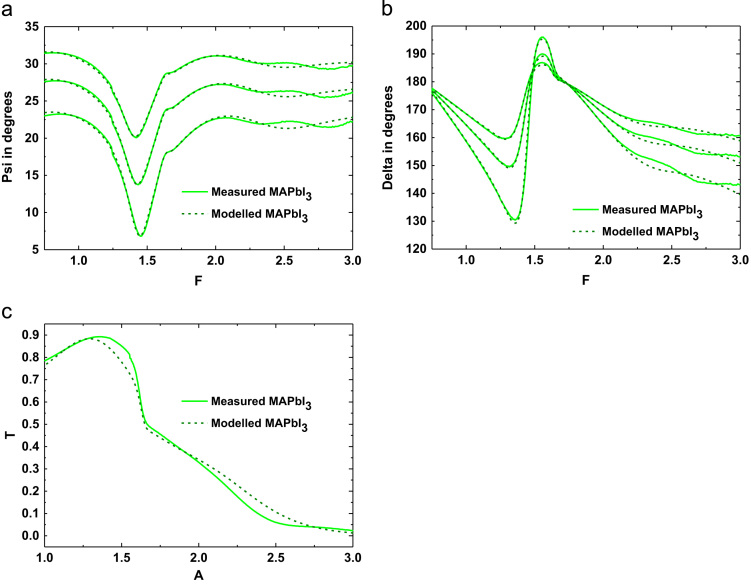
Modelled (dash lines) and experimental (solid lines) (a) amplitude component *Ψ*; (b) phase difference *Δ* and (c) Transmission *T* of CH_3_NH_3_PbI_3_ perovskite film on TiO_2_ coated borosilicate glass substrate.

**Fig. 5:**
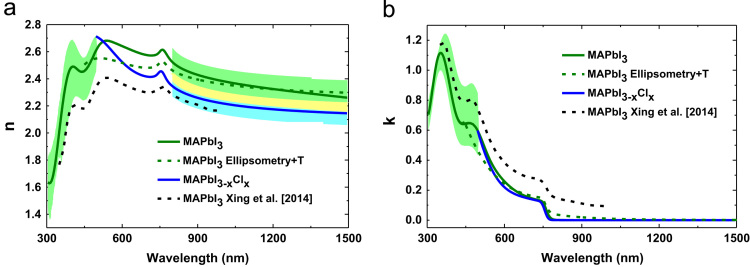
The modelled (a) refractive index *n* and (b) extinction coefficient *k* of CH_3_NH_3_PbI_3_ and CH_3_NH_3_PbI_3−*x*_Cl_*x*_ thin films compared to results reported by Xing et al. [8] (dashed line).

Fig. 4 shows the experimental and modelled amplitude component *Ψ*; phase difference *Δ*; transmission *T* of the CH_3_NH_3_PbI_3_ perovskite which are used to determine the optical constants as a supplementary method. The CH_3_NH_3_PbI_3_ perovskite film thickness has been determined to be 173 nm and 40 nm surface roughness with 89% void. The optical constants *n* and *k* deduced from this modelling are compared with other data sets in Fig. 5(a) and (b) respectively.

## Figures and Tables

**Table 1:** Parameters for Optical Models of *ε*_2_ for borosilicate glass and glass. *A*, *E* and *B* represent the amplitude, centre energy and broadening of Gaussian oscillator. *A*_*n*_ and *B*_*n*_ are parameters in Cauchy dispersion for refractive index *n*.

Oscillators (borosilicate glass)	*A* (eV^2^)	*E* (eV)	*B* (eV)	Oscillators (Glass)	*A*_*n*_ (dimentionless)	*B*_*n*_ (μm^2^)
Gaussian	1.49	5.88	0.85	Cauchy	1.4373	0.0058
Gaussian	2.40	5.64	0.70			

**Table 2:** Parameters for Optical Models of *ε*_2_ for TiO_2_/Glass. *A*, *E*, *B* and *E*_g_ represent the amplitude, centre energy, broadening and bandgap of the oscillator.

Oscillators	*A* (eV^2^)	*E* (eV)	*B* (eV)	*E* (eV)_g_
Tauc–Lorentz	259.65	3.98	2.19	3.44

**Table 3:** Parameters for Optical Models of *ε*_*2*_ for CH_3_NH_3_PbI_3_ film.

Oscillators	*A* (eV^2^)	*E* (eV)	*B* (eV)	WL (eV)	WR (eV)	AL (eV)	AL (eV)
PSTRI	0.65	1.64	0.02	0.22	5.12	0.01	0.82
PSTRI	2.73	2.81	0.21	0.93	0.01	0.51	0.11
Gaussian	0.39	2.48	0.33				
Gaussian	3.73	3.35	0.82				
Gaussian	1.57	4.51	1.65				

**Table 4:** Parameters for Optical Models of *ε*_*2*_ for CH_3_NH_3_PbI_3−*x*_Cl_*x*_ film.

Oscillators	*A* (eV^2^)	*E* (eV)	*B* (eV)	WL (eV)	WR (eV)	AL (eV)	AL (eV)
PSTRI	0.59	1.66	0.02	0.04	4.58	0.44	0.95
PSTRI	5.86	3.54	0.27	1.36	2.28	0.86	0.01
